# Exposure variables in veterinary epidemiology: are they telling us what we think they are?

**DOI:** 10.3389/fvets.2024.1442308

**Published:** 2024-07-30

**Authors:** Audrey Ruple, Jan M. Sargeant, Annette M. O’Connor, David G. Renter

**Affiliations:** ^1^Department of Population Health Sciences, VA-MD College of Veterinary Medicine, Virginia Tech, Blacksburg, VA, United States; ^2^Department of Population Medicine, Ontario Veterinary College, University of Guelph, Guelph, ON, Canada; ^3^Department of Large Animal Clinical Sciences, College of Veterinary Medicine, Michigan State University, East Lansing, MI, United States; ^4^Center for Outcomes Research and Epidemiology, College of Veterinary Medicine, Kansas State University, Manhattan, KS, United States

**Keywords:** exposure variables, variable selection, observational studies, veterinary epidemiology, causation

## Abstract

This manuscript summarizes a presentation delivered by the first author at the 2024 symposium for the Calvin Schwabe Award for Lifetime Achievement in Veterinary Epidemiology and Preventive Medicine, which was awarded to Dr. Jan Sargeant. Epidemiologic research plays a crucial role in understanding the complex relationships between exposures and health outcomes. However, the accuracy of the conclusions drawn from these investigations relies upon the meticulous selection and measurement of exposure variables. Appropriate exposure variable selection is crucial for understanding disease etiologies, but it is often the case that we are not able to directly measure the exposure variable of interest and use proxy measures to assess exposures instead. Inappropriate use of proxy measures can lead to erroneous conclusions being made about the true exposure of interest. These errors may lead to biased estimates of associations between exposures and outcomes. The consequences of such biases extend beyond research concerns as health decisions can be made based on flawed evidence. Recognizing and mitigating these biases are essential for producing reliable evidence that informs health policies and interventions, ultimately contributing to improved population health outcomes. To address these challenges, researchers must adopt rigorous methodologies for exposure variable selection and validation studies to minimize measurement errors.

## 1 Introduction

John Snow, considered the father of modern epidemiology, published his conclusions regarding the Broad Street pump being the source of the Cholera epidemic in the Soho district of London in 1855. In terms of scientific advances this is still a relatively modern development and epidemiology is thus a relatively young science. To put this in perspective, we are equidistant from John Snow’s publication “On the mode of communication of cholera” now as he was from Sir Isaac Newton’s publication about the laws of motion (1687) at the time he presented that publication.

Given the foundations of this branch of science and the most pressing health-related issues facing human populations at the time, it is no surprise that the early developments in the field of epidemiology were rooted in determining the cause(s) of infectious diseases. In this model, causal factors are those that are responsible for health impacts or modifications of health and each factor that contributes to disease occurrence is considered a component cause of disease. Any combination of factors that produce disease are considered a sufficient cause of disease, and causal factors that are required for the disease to develop are termed necessary causes. However, as we have moved from studying infectious causes of disease to non-infectious disease outcomes, such as cancer and aging in both humans and other animal species, we have increased the complexity of exposure measurement within the field. This is because with non-infectious outcomes there may be no necessary cause for a particular health outcome. In fact, any single component cause may only make a small contribution to the disease etiology. This perspective aims to elucidate the importance of appropriate selection of exposure variables within the field of veterinary epidemiology, though many of the concepts apply to human populations as well.

## 2 Challenges with exposure variables

Rothman and Greenland (1) described the concept of causation due to multiple component causes as being an incomplete causal mechanism unless or until all of the component conditions or events that are necessary for the outcome to occur have reached a set of minimal conditions or thresholds. Thus, each of those components must be accurately measured to determine causality. An additional complexity is that most diseases can be caused by more than one causal mechanism, a concept called multicausality, and each of these mechanisms involves the collective action of a multitude of component causes (1). Knowledge of which components are part of the multiple component causes and how they should be measured is necessary prior to occurrence of the outcome of interest in order to determine causality.

When measuring exposures, it is also important to consider the timing of the exposure on the individual or population in terms of when the exposure occurs in relation to the individual’s development or life stage. This is important because the timing of the exposure can cause tremendous variability in the outcomes that may occur. An excellent example is the exposure to the steroidal alkaloid, cyclopamine, in sheep during pregnancy. Ewes can become exposed to this potent teratogen through ingestion of the plant *Veratrum californicum* resulting in synophthalmia (cyclopia) formation in the embryonic lamb. However, cyclopamine is rapidly eliminated from the ewe and ingestion of the plant only on gestational days 13 or 14 results in craniofacial malformations being exhibited (2).

In addition to the timing of exposure in relation to the individual’s development, duration of exposure may also be associated with outcomes. In a prospective human birth cohort study conducted in Cincinnati, Ohio, early life exposure to traffic-related air pollution was associated with wheezing regardless of the age at which exposure occurs (3). However, increased risk for asthma was only identified in children exposed to traffic-related air pollution from birth to the age of seven (3). This illustrates that, even within the same population cohort, the duration of time exposed to the same exposure risk did influence disease occurrence.

Another complication is that many of the observational studies used in veterinary epidemiology are retrospective. However, it is not always possible to measure exposure variables retrospectively, as it is often the case that there are no measurable indicators of past exposures. For instance, dietary intake during childhood has been shown to affect adult risk of breast cancer in human females (4), but there are few adult individuals who have detailed descriptions of the types and amounts of foods they consumed as toddlers.

The total number of exposures of interest have increased considerably, too. In a recently published manuscript by Sargeant et al. (5), the authors evaluated 200 observational studies published in the veterinary literature between 2020 and 2022. The number of variables assessed during the screening step in these studies averaged over 20, with a maximum of more than 175. The average number of independent variables evaluated in the final models used in the studies was approximately 14.

The exposure variables being examined themselves have also become much more complex. For instance, food selections for companion animals have become more diverse (6), and different diet types have been associated with different health outcomes (7, 8). Environmental risk factors being examined in relation to health outcomes in animals include those related to the natural environment (9), built environment (10), and the chemical environment (11). Researchers are examining the role that psychosocial (12) and cognitive states (13) play in health outcomes in animals as well. Of course, we also are learning more about the role that genetic predispositions play in the outcome of disease, especially cancers (14), in animal species.

This increasing complexity and numeracy of exposures of interest has likely contributed to an increase in errors related to measurement of exposures (15, 16). It is thought that inaccurate exposure measurements are one of the main sources of bias in epidemiologic research. The magnitude of this bias is likely underappreciated (16). For instance, if we have a well measured variable that correlates with the true exposure of interest with a correlation coefficient of 0.7, we might consider that to be an acceptably strong relationship between the two variables. However, in this instance if we observe a risk ratio of 1.7 in our exposure variable with a correlation coefficient of 0.7, it would indicate that the true risk ratio associated with the exposure of interest is 3.0, nearly two-fold higher than what was measured. Of course, exposure estimates can be either under- or overestimated when measurement errors occur (17).

With the era of veterinary medical “Big Data” having begun (18), one might assume that measurement errors can be overcome by the use of enormous datasets with large numbers of observations. This assumption likely originates from the probability theory known as the law of large numbers wherein by taking the average of an increasing number of random observations sampled from a population it allows for convergence on the true value of the mean. However, measurement errors impact epidemiologic data analyses in several ways, including creating bias in, and affecting the precision of, the exposure effect estimate (17). Thus, a larger sample size will not necessarily move exposure effect estimates closer to their real values and may affect the precision of the estimate, but not the bias resulting in a very precise, but biased estimate. So a larger sample size might be able to compensate for the loss in precision that is caused by measurement error, but the bias created when the reliability of the measurement is low may need a 50-fold or more increase in sample size in order to compensate for the error (19, 20).

It is not uncommon for veterinary researchers to use proxy variables in lieu of directly measuring the true variable of interest. One type of proxy measure that is used with some frequency in epidemiologic research is distance. That is to say that we use the *distance* from an exposure of interest as a proxy measure for the *amount* of exposure. In many cases, investigators are able to measure distance from the exposure with a high degree of accuracy, but the true amount of exposure may not always be equal at equal distances from the source of exposure. For instance, a virus or fine particulate matter that is dispersed through the air and travels from a source of exposure like a silver mine (21) or a poultry house (22) does not travel uniformly in all directions away from the source of exposure. Factors such as wind direction and speed, the deposition process, and pathogen decay rate must be considered in order for true exposure to be estimated. Similarly, all animals in a closed barn may not receive the same exposure from an airborne pathogen due to differences in air flow within the building based on location of fans and doors and variables such as temperature and humidity. However, distance is regularly used as a proxy measure for exposure without accounting for variables that might differentially impact the way in which distance from a source of exposure should be interpreted in both human (23) and animal (24) health research.

It is also not uncommon for veterinary researchers to create variables to define exposures of interest. For example, there have been several studies that have examined the effect of brachycephaly, or a shortened skull shape, on health outcomes in dogs (25–27). However, there is not a standardized definition of the term brachycephaly being used across these studies. One study (25) used morphometric measurements to define dogs as brachycephalic, another (26) used a list of 13 dog breeds to define their brachycephalic cohort, and a third (27) used a list of more than 30 dog breeds to define their brachycephalic cohort, and that list did not incorporate all of the 13 breeds included in the previous study. Thus, the same exposure variable was ostensibly being examined, but on close inspection it becomes apparent that though the same label is being affixed, the term does not mean the same thing in each of these instances. This means that at least some of the animals or even entire breeds being studied must be misclassified when we compare results across studies.

## 3 Proposed solutions

Given that inaccurate exposure measurements are one of the main sources of bias in epidemiologic research, it seems prudent that we, as a discipline, make every effort to reduce the impact on our understanding of health. One of the most straightforward ways we can do this is by directly measuring exposure variables of interest. Foregoing the use of proxy measurements whenever feasible and realistic to do so will decrease bias and increase the accuracy of our exposure measurements. This will in turn allow us to observe risk ratios that are closer to the true effect and will enhance our understanding of disease etiologies.

When it is not possible to directly measure the exposure variable of interest, it is imperative that rational proxy measurements are used. Thoughtfully considering how the proxy measure may vary from the true exposure variable and taking those variables into account is crucial. Furthermore, it is imperative that the process through which the proxy variable was decided upon by the investigators be described in the methods section of the report associated with the work. Transparency around the decision-making process is critical so that readers can evaluate and determine how close a proxy measurement is to the true variable of interest.

Directed acyclic graphs (DAGs) or causal diagrams can also be used for selecting appropriate exposure variables as they provide a clear representation of the assumed causal relationships between variables. By mapping out these relationships, DAGs help to identify and distinguish between confounders, mediators, and colliders, thus preventing biased estimates of the exposure-outcome association (28). When used to guide the selection of variables to control for, they help to ensure that the chosen variables isolate the causal effect of the exposure on the outcome, rather than introducing bias or masking the true relationship.

Further, we must be consistent in our use of defined exposures. Using similar terminology with different inclusion criteria across studies makes research replication difficult, if not impossible. Our profession has a strong history of successfully using consensus statements to provide our community with information about topics as varied as the diagnosis and treatment of diseases to reporting guidelines for use when conducting research (29–34). Consensus statements also can be used to define exposure variables that can be uniformly applied across research endeavors.

Lastly, failure to recognize the impact of poorly measured exposure variables should not be tolerated. They should, in fact, be considered a serious flaw in research proposals and manuscripts submitted for publication. Erroneous measurements can lead to biased results that may not be sufficiently understood, even when they are recognized by the researchers. Several methods of quantitative bias analysis and “good practices” for their application have been developed (35). Acknowledging the presence of errors in the measurement of exposure variables in the discussion section of a manuscript should not be considered an adequate or acceptable practice.

## Data availability statement

The original contributions presented in the study are included in the article/supplementary material, further inquiries can be directed to the corresponding author.

## Author contributions

AR: Conceptualization, Formal analysis, Writing – original draft, Writing – review & editing. JS: Conceptualization, Writing – review & editing. AO'C: Conceptualization, Writing – review & editing. DR: Conceptualization, Writing – review & editing.
